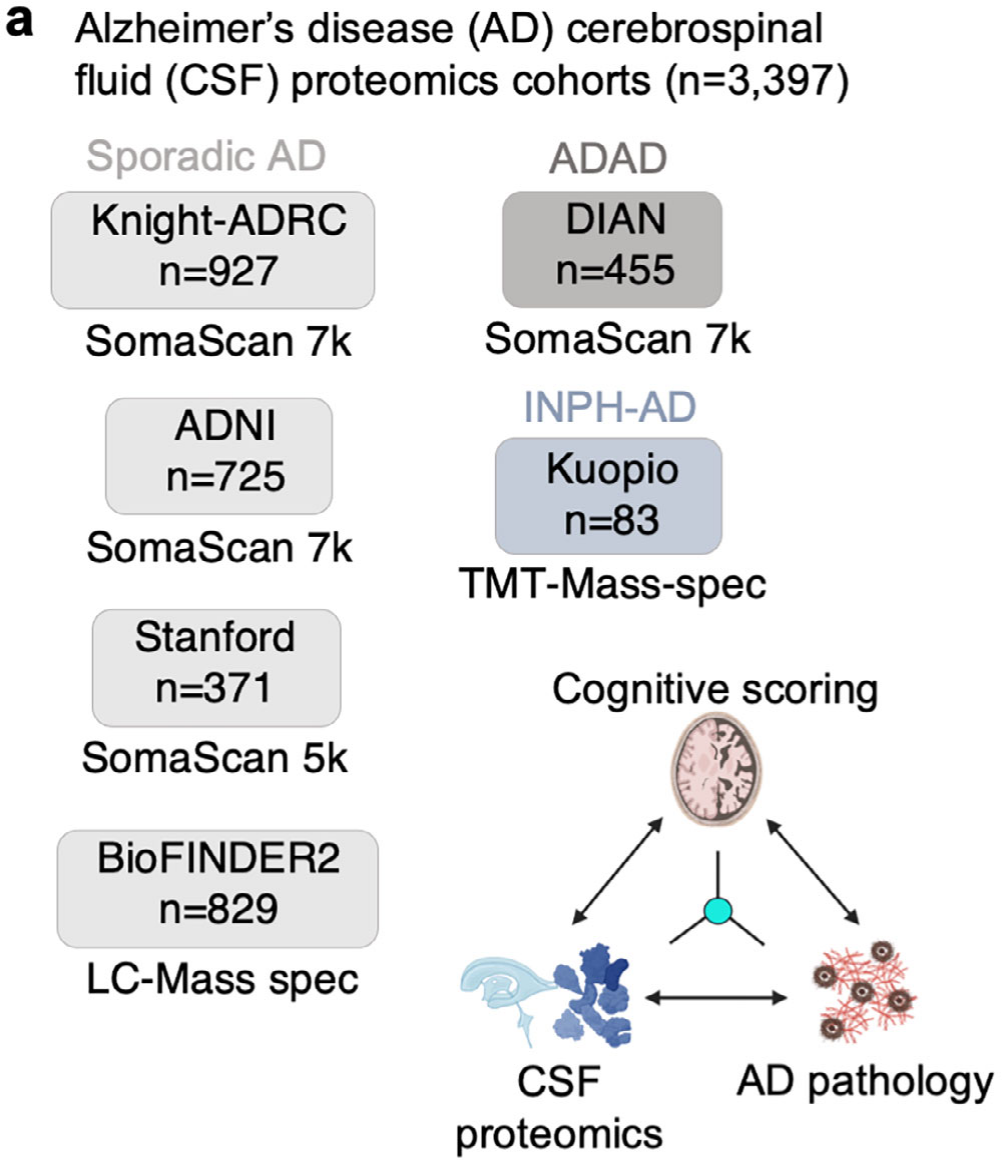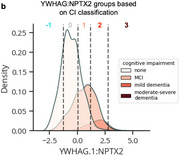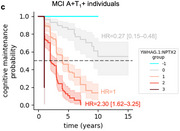# A cerebrospinal fluid synaptic protein biomarker for prediction of cognitive resilience versus decline in Alzheimer's disease

**DOI:** 10.1002/alz70856_102349

**Published:** 2025-12-25

**Authors:** Hamilton Se‐Hwee Oh, Deniz Yagmur Urey, Linda Karlsson, Zeyu Zhu, Yuanyuan Shen, Amelia Farinas, Jigyasha Timsina, Michael R Duggan, Jingsha Chen, Ian H Guldner, Nader Morshed, Chengran Yang, Daniel Western, Muhammad Ali, Yann Le Guen, Alexandra N. Trelle, Sanna‐Kaisa Herukka, Tuomas Rauramaa, Mikko Hiltunen, Anssi Lipponen, Antti J Luikku, Kathleen L. Poston, Elizabeth C. Mormino, Anthony D. Wagner, Ted N. Wilson, Divya Channappa, Ville Leinonen, Beth Stevens, Alexander J. Ehrenberg, Rebecca F. Gottesman, Josef Coresh, Keenan A. Walker, Henrik Zetterberg, David A. A. Bennett, Nicolai Franzmeier, Oskar Hansson, Carlos Cruchaga, Tony Wyss‐Coray

**Affiliations:** ^1^ Icahn School of Medicine at Mount Sinai, New York, NY, USA; ^2^ Wu Tsai Neurosciences Institute, Stanford University, Stanford, CA, USA; ^3^ Stanford University, Stanford, CA, USA; ^4^ Clinical Memory Research Unit, Department of Clinical Sciences, Lund University, Lund, Sweden; ^5^ Institute for Stroke and Dementia Research (ISD), University Hospital, LMU Munich, Munich, Bavaria, Germany; ^6^ Washington University in St. Louis, School of Medicine, St. Louis, MO, USA; ^7^ Washington University School of Medicine, St. Louis, MO, USA; ^8^ NeuroGenomics and Informatics Center, Washington University School of Medicine, St. Louis, MO, USA; ^9^ Department of Psychiatry, Washington University School of Medicine, St. Louis, MO, USA; ^10^ Laboratory of Behavioral Neuroscience, National Institute on Aging, Intramural Research Program, Baltimore, MD, USA; ^11^ Department of Epidemiology, Johns Hopkins University Bloomberg School of Public Health, Baltimore, MD, USA; ^12^ Department of Neurology and Neurological Sciences, Stanford, CA, USA; ^13^ Boston Children's Hospital, Boston, MA, USA; ^14^ Quantitative Sciences Unit, Department of Medicine, Stanford University School of Medicine, Stanford, CA, USA; ^15^ Department of Neurology and Neurological Sciences, Stanford University School of Medicine, Stanford, CA, USA; ^16^ Stanford University School of Medicine, Stanford, CA, USA; ^17^ Kuopio University Hospital, Kuopio, Finland; ^18^ University of Eastern Finland, Kuopio, Finland; ^19^ Department of Neurology and Neurological Sciences, Stanford University, Stanford, CA, USA; ^20^ Broad Institute of MIT and Harvard, Cambridge, MA, USA; ^21^ Boston Children's Hospital, Boston, MA, USA; ^22^ Memory and Aging Center, Weill Institute for Neurosciences, University of California, San Francisco, San Francisco, CA, USA; ^23^ Helen Wills Neuroscience Institute, University of California, Berkeley, Berkeley, CA, USA; ^24^ Innovative Genomics Institute, Berkeley, CA, USA; ^25^ National Institute of Neurological Disorders & Stroke, Bethesda, MD, USA; ^26^ Departments of Population Health and Medicine, New York University Grossman School of Medicine, New York, NY, USA; ^27^ Department of Psychiatry and Neurochemistry, Institute of Neuroscience and Physiology, the Sahlgrenska Academy, University of Gothenburg, Molndal, Sweden; ^28^ Hong Kong Center for Neurodegenerative Diseases, Hong Kong, Science Park, China; ^29^ University College London, London, United Kingdom, London, United Kingdom; ^30^ UK Dementia Research Institute at UCL, London, United Kingdom; ^31^ Rush Alzheimer's Disease Center, Chicago, IL, USA; ^32^ University of Gothenburg, The Sahlgrenska Academy, Institute of Neuroscience and Physiology, Psychiatry and Neurochemistry, Gothenburg, Sweden; ^33^ Munich Cluster for Systems Neurology (SyNergy), Munich, Bavaria, Germany; ^34^ Institute for Stroke and Dementia Research (ISD), LMU University Hospital, LMU, Munich, Bavaria, Germany; ^35^ Clinical Memory Research Unit, Department of Clinical Sciences, Lund University, and Memory Clinic, Skåne University Hospital, Malmö, Sweden; ^36^ Skåne University Hospital, Malmö, 21428 Skåne, Sweden

## Abstract

**Background:**

Rates of cognitive decline in Alzheimer's disease (AD) are extremely heterogeneous, with symptom onset occurring between ages 40‐100 years and conversion from mild cognitive impairment (MCI) to AD dementia occurring in 2‐20 years. Biomarkers for amyloid‐beta (Aβ) and tau proteins, the hallmark AD pathologies, have improved diagnosis and drug development but explain only 20‐40% of the variance in AD‐related cognitive impairment (CI).

**Method:**

To discover additional biomarkers of CI in AD, we perform cerebrospinal fluid (CSF) proteomics on 3,397 individuals from six major prospective AD case‐control cohorts. Synapse proteins emerge as the strongest correlates of CI, independent of Aβ and tau.

**Result:**

Using machine learning, we derive the CSF YWHAG:NPTX2 synapse protein ratio, which explains 27% of the variance in CI beyond CSF PTau181:Aβ42, 10% beyond tau PET, and 28% beyond CSF NfL, GAP43, and Ng in Aβ‐ and phosphorylated tau‐ positive (A+T_1_+) individuals. We find YWHAG:NPTX2 also increases with normal aging and at a faster rate in *APOE4* carriers and autosomal dominant‐AD mutation carriers. For prognosis, we define YWHAG:NPTX2 thresholds to stratify A+T_1_+ individuals into five groups that track with future cognitive resilience versus decline. Most notably, among A+T_1_+ MCI individuals, those in the predicted cognitively normal group have a 73% reduced risk of cognitive decline, while those in the predicted dementia group have a 2.3 times increased risk, after adjusting for CSF PTau181:Aβ42, CSF NfL, CSF Ng, CSF GAP43, age, *APOE4*, and sex. Lastly, we develop a plasma proteomic signature of CI, which we evaluate in 13,401 samples, that partly recapitulates CSF YWHAG:NPTX2.

**Conclusion:**

Overall, our findings underscore CSF YWHAG:NPTX2 and the corresponding plasma signature as robust prognostic biomarkers for AD onset and progression beyond gold‐standard biomarkers of Aβ, tau, and neurodegeneration and implicate synapse dysfunction as a core driver of AD dementia.